# Molecular surveillance of anti-malarial drug resistance genes in *Plasmodium falciparum* isolates in Odisha, India

**DOI:** 10.1186/s12936-022-04403-3

**Published:** 2022-12-24

**Authors:** Ramakanta Rana, Nikhat Khan, Sonali Sandeepta, Sanghamitra Pati, Aparup Das, Madhusmita Bal, Manoranjan Ranjit

**Affiliations:** 1 Molecular Epidemiology Laboratory, ICMR-Regional Medical Research Centre, Bhubaneswar, Odisha 751023 India; 2 Division of Vector-Borne Diseases, ICMR-National Institute of Research in Tribal Health, Jabalpur, Madhya Pradesh India

**Keywords:** *Plasmodium falciparum*, Drug-resistant markers, Chloroquine, Sulphadoxine-Pyrimethamine

## Abstract

**Background:**

Despite significant progress in eliminating malaria from the state of Odisha, India, the disease is still considered endemic. Artesunate plus sulfadoxine-pyrimethamine (AS + SP) has been introduced since 2010 as first-line treatment for uncomplicated *Plasmodium falciparum* malaria. This study aimed to investigate the prevalence of mutations associated with resistance to chloroquine (CQ), sulfadoxine-pyrimethamine (SP), and artesunate (ART) in *P. falciparum* parasites circulating in the state.

**Methods:**

A total of 239 isolates *of P. falciparum* mono infection were collected during July 2018-November 2020 from the four different geographical regions of the state. Genomic DNA was extracted from 200 µL of venous blood and amplified using nested polymerase chain reaction. Mutations on gene associated with CQ (*Pfcrt* and *Pfmdr1*) were assessed by PCR amplification and restriction fragment length polymorphism, artemisinin (*Pfk13)* gene by DNA sequencing and SP (*Pfdhfr* and *Pfdhps*) genes by allele-specific polymerase chain reaction (AsPCR).

**Results:**

The point mutation in *Pfcrt* (K76**T**) was detected 2.1%, in *Pfmdr1* (N86**Y**) 3.4%, and no mutations were found in *Pfkelch13* propeller domain. Prevalence of *Pfdhfr, Pfdhps* and *Pfhdfr-Pfdhps* (two locus) gene mutations were 50.43%, 47.05% and 49.79% respectively. The single, double, triple and quadruple point mutations in *Pfdhfr* gene was 11.2%, 8.2%, 17.2% and 3.4% while, in *Pfdhps* gene was 10.9%,19.5%, 9.5% and 2.7% respectively. Of the total 13 haplotypes found in *Pfdhfr*, 8 were detected for the first time in the state and of the total 26 haplotypes found in *Pfdhps*, 7 were detected for the fisrt time in the state. The linked quintuple mutation *Pfdhfr* (N51**I**-C59**R**-S108**N**)-*Pfdhps* (A437**G**-K540**E**) responsible for clinical failure (RIII level of resistance) of SP resistance and A16**V**-S108**T** mutation in *Pfdhfr* responsible for cycloguanil was absent.

**Conclusion:**

The study has demonstrated a low prevalence of CQ resistance alleles in the study area. Despite the absence of the *Pfkelch13* mutations, high prevalence of *Pfdhfr* and *Pfdhps* point mutations undermine the efficacy of SP partner drug, thereby threatening the *P. falciparum* malaria treatment policy. Therefore, continuous molecular and in vivo monitoring of ACT efficacy is warranted in Odisha.

## Background

Malaria caused by *Plasmodium* species occurs mainly in poor tropical and sub-tropical regions of the world. Of the five species causing human malaria, *Plasmodium falciparum* is the most lethal and accounts for more than 90% of the world's malaria deaths [1]. Although malaria mortality has been reduced by over a quarter around the world, its transmission still occurs in 99 countries. In 2020, an estimated 241 million malaria cases and 627,000 malaria deaths have occurred worldwide [2]. In Southeast Asia, India alone accounts for 85.2% of malaria cases and 2% of global malaria deaths [3]. While in India, the state of Odisha, with 4% of the total population of the country, accounted for 32.4% of the total malaria cases, 32.02% of *P. falciparum* cases, and 9.7% of malaria deaths in 2020 (NVBDCP, Govt. of India).

In absence of an effective vaccine, chemotherapy and chemoprophylaxis remain the principal means to minimize the mortality and morbidity burden due to malaria. As in other malaria-endemic countries of the world, chloroquine (CQ) was used in the national programme in India since 1961 as the first-line treatment for uncomplicated malaria for a prolonged period because of its safety profile and cost-effectiveness. After sustained use, the resistance of *P. falciparum* to CQ emerged first time in India in 1973 in the Karbi-Anglong district of Assam that subsequently spread across the country [4, 5]. Accordingly, the Indian drug policy was changed and the sulfadoxine-pyrimethamine (SP) combination was introduced in 1995 as a second-line treatment [6]. However, in 2004 the World Health Organization (WHO) technical advisory group recommended the use of combination anti-malarial therapy, particularly with artemisinin derivatives, in member countries for treating *P. falciparum* to delay the emergence of drug resistance. Consequently, artemisinin-based combination therapy (ACT) i.e. using artesunate + sulfadoxine-pyrimethamine (AS-SP) was first introduced in 2004 in CQ resistant areas and then implemented in the rest of the country in 2010 as the first-line treatment of *P. falciparum* malaria [7].

The effective implementation of any drug policy needs continuous monitoring of drug-resistant parasites in the field to determine the spread of resistance over wide areas. Since the identification of drug-resistant *P. falciparum* strains by in vitro assay and standard 28-day in vivo efficacy study are cumbersome, molecular markers have been proposed as an alternative tool to monitor resistance [8]. The point mutation in *P. falciparum* CQ transporter (*Pfcrt*) gene (K76**T**) and *P. falciparum* multidrug-resistance1 (*Pfmdr1*) gene (N86**Y**) have been found to be associated with CQ resistance [9, 10]. Resistance to SP drug combination has been shown to occur due to the point mutations in the *P. falciparum* dihydrofolate reductase (*Pfdhfr*) gene (A16**V**, C50**R**, N51**I**, C59**R**, S108**T**/**N**, and I164**L**) and *P. falciparum* dihydropteroate synthase (*Pfdhps*) gene (S436**A/F**, A437**G**, K540**E**, A581**G**, and A613**S/T**) [11]. Multiple mutation combinations of both *Pfdhps* and *Pfdhfr* were responsible in varying the level of SP resistance. While, mutations in the propeller domain of the Kelch-13 protein encoded by the *P. falciparum Pfk13* gene have been associated with delayed parasite clearance due to resistance to artemisinin (ART) in the Greater Mekong Sub-regions of Southeast Asia and sub-Saharan regions of Africa [12–15].

India has pledged to eliminate malaria in the entire country by 2030 [16]. To achieve the target, wide coverage of molecular data on anti-malarial drug resistance is essential for proper implementation of drug treatment policy. Hence, the present study was undertaken to assess the prevalence of *Pfcrt* K76**T** and *Pfmdr1* N86**Y** (responsible for chloroquine resistance), mutations of *Pfdhfr* and *Pfdhps* genes responsible for SP drug resistance and *P. falciparum Pfk13* polymorphism associated with artemisinin (ART) treatment failure on *P. falciparum* isolates in Odisha, between 2018 and 2020, after ten years of the introduction of new drug policy. The data from the study could contribute to baseline information on the distribution of anti-malarial drug resistance, particularly in Odisha prior to malaria elimination.

## Methods

### Study setting and sample collection

**Fig.1:**
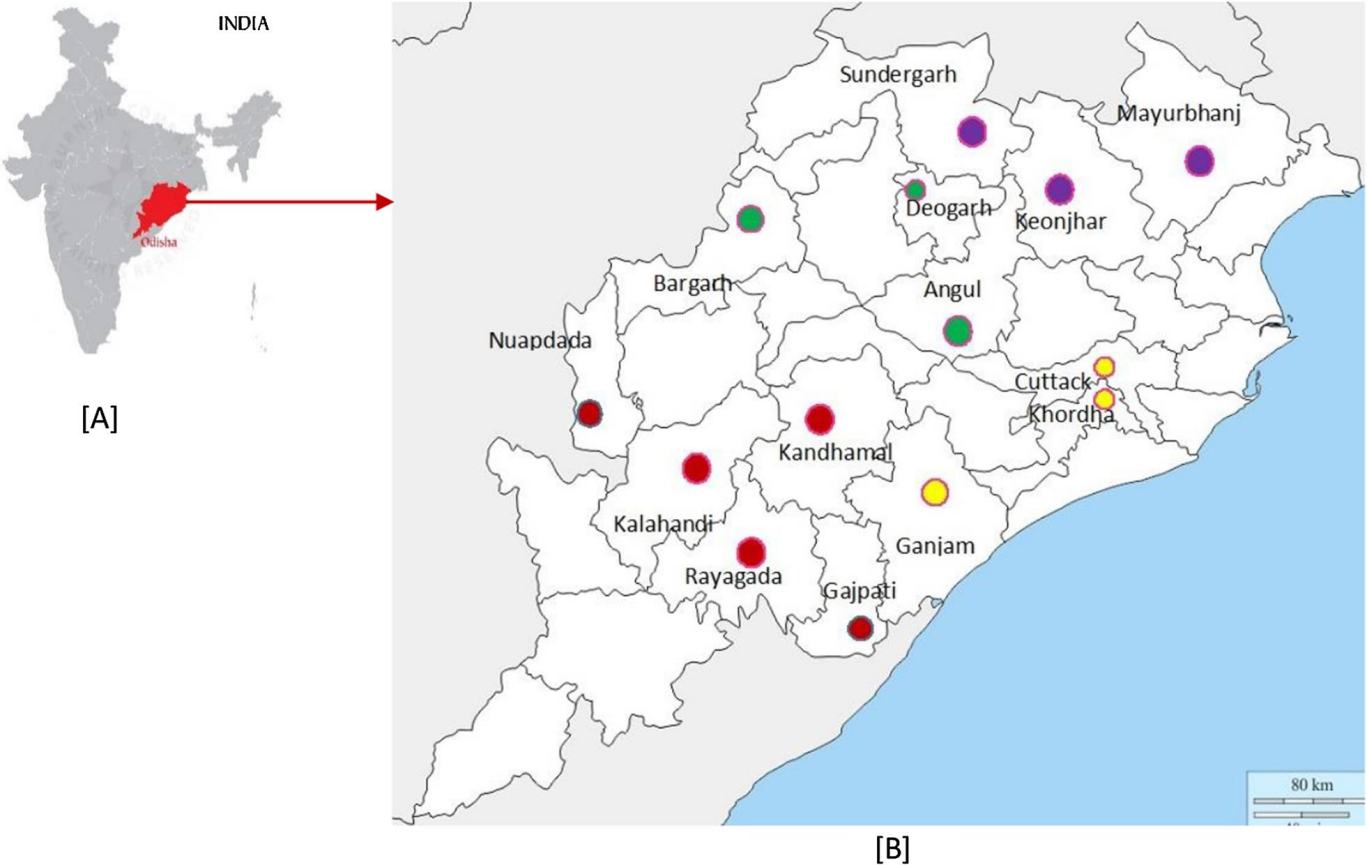
Map of India **A** and district of Odisha **B** indicating the study districts of four geographical regions. The base maps were taken from the website: https://www.burningcompass.com/countries/India/odisha-on-India-map-hd.html and was modified using Microsoft word and paint. The studied fourteen districts of the four different geographical regions marked by four distinct marks (i) Northern Plateau (purple), (ii) Eastern Ghat (red), (iii) Central Tableland (green) and (iv) Coastal Belt (yellow)

This study was conducted between July 2018 to November 2020 among the patients attending government health facilities in different districts representing four geographical regions of the state (Eastern Ghat: Raygada, Kalahandi, Nuapada, Kandhamal, Gajapati, Northern Plateau: Mayurbhanj, Sundergarh, and Keonjhar, Central Tableland: Bargarh, Angul, Deogarh and, Coastal Belt: Cuttack, Khorda, and Ganjam) as shown in Fig. 1. Based on the overall annual parasitic index (API) of the districts as reported by NVBDCP, Odisha, 2016, the Eastern Ghat and Northern Plateau can be categorized as highly endemic (API > 10), the Central Tableland (API 5–10) as moderately endemic and Coastal Belt districts are very low endemic (API < 0.5). As per Indian drug policy, ACT has been used as a treatment for *P. falciparum* infection, and chloroquine + primaquine was used for *P. vivax* infection. As per the available literature the prevalence of CQ and SP drug-resistant haplotypes was high in the districts of Northern plateau, Eastern Ghat, moderate in the districts of Central Tableland and low in the districts of Coastal Belt [17, 18]. Suspected malaria cases were screened by the WHO evaluated *Pf* PAN Ag Rapid Diagnostic Test Kit (RDT) (SD-Biosensor, India) using finger-prick blood [19]. Approximately one mL of venous blood was collected in BD Vacutainer^®^EDTA vial from individuals found to be positive for malaria. The blood samples collected in the field were preserved at 4^0^C at the local hospital and transported to the Indian Council of Medical Research (ICMR)-Regional Medical Research Centre (RMRC) Bhubaneswar laboratory within 24 h, maintaining a cold chain for further molecular analysis.

### Diagnosis and speciation by PCR

Parasite genomic DNA was extracted from 200 μL of EDTA blood samples using QIAamp Blood DNA mini kit (QIAGEN, Germany) according to the manufacturer's instructions and eluted in 50 μL TE buffer. Nested PCR (nPCR) was performed to confirm the diagnosis and identification of the species using the species specific primers targeting 18SrRNA and cycling parameters as described by Snounou et al. [20] in a thermal cycler (Agilent Sure Cycler 8800, USA). Briefly, the primary PCR was performed on a 25 μL reaction mixture that contained 0.2 U of Taq DNA polymerase (GCC Biotech, India), 0.2 mM each dNTP (HIMEDIA Laboratories, India), 0.4 mM each primer (GCC Biotech, India) and 2.0 mM MgCl_2_ (GCC Biotech, India). The reaction was allowed to proceed for 35 cycles after an initial denaturation at 94^0^ for 1 min, annealing at 50^0^ for 1 min, and extension at 72^0^ for 1 min; final extension was at 72^0^ for 10 min. The nested PCR reaction condition was the same as primary PCR except for the annealing temperature, 55^0^. PCR products were visualized under UV light following the electrophoresis on 2.0% (w/v) ethidium bromide stained agarose gel, and images were captured using a gel documentation system (Alpha Imager, USA). Previously diagnosed *Plasmodium* species specific DNA was used as positive control and genomic DNA extracted from uninfected individuals was used as negative control.

### Analysis of* Pfcrt, Pfmdr1* &* Pfk13* genes

Genotyping of the resistant markers *Pfcrt* K76**T** and *Pfmdr1* N86**Y** was carried out by PCR-Restriction fragment length polymorphism (RFLP) using the genomic DNA isolated from all the enrolled samples (n = 239) followed by sequencing for *Pfcrt* and *Pfmdr1* haplotype analysis. Briefly, PCR protocol and the primers (Table 1) as described elsewhere [17] amplified the 264 bp *Pfcrt* gene spanning the codon region from 72 to 76, 78, 97 and the 603 bp *pfmdr1* gene with codons 86,130,184. The PCR products were digested with Type II restriction digestion enzyme *Apo I* for detection of the *Pfcrt* sensitive/resistant genotype and *Afl III* for *Pfmdr1* sensitive/resistant genotype. The *Apo I* digests the 264 bp *Pfcrt* PCR product into 128 bp and 136 bp fragments in case of the CQ wild (sensitive) allele, but the mutant allele associated with CQ resistance remains undigested. Similarly, 603 bp of *Pfmdr1* PCR product when treated with *Afl III* the mutant (resistant) allele digested into 353 bp and 253 bp fragments, while the CQ/multidrug-sensitive (wild) genotypes remain undigested. The *Pfkelch13* gene fragment was amplified by nested PCR protocols reported previously with modifications [12].

**Table 1:** Details of the primers, restriction enzymes and cycling conditions used for *Pfcrt* and *Pfmdr1* RFLP analysis

Gene	Primer sequence (5'-3')	Product size (bp) & Restriction enzyme		PCR cycling conditions
*Pfcrt*-F	GGCTCCACGTTTAGGTGGA	264	CQS-128 & 136 bp	Initial: 95 ℃-5 min **30 cycles** 95 ℃-1 min 48 ℃-45 s 72 ℃-2 min Final: 72 ℃-10 min
*Pfcrt*-R	TGAATTTCCCTTTTTATTTCCAAA	*Apo I*	CQR-Undigested	
*Pfmdr1*-F	ATGGGTAAAGAGCAGAAAGA	603 bp	CQR-253 & 350 bp	Initial: 95 ℃-5 min, **30 cycles** 95 ℃-1 min 56 ℃-45 s 72 ℃-2 min Final: 72 ℃-10 min
*Pfmdr1*-R	AACGCAAGTAATACATAAAGTCA	*Afl III*	CQS-Undigested	

In case of *Pfcrt,* 212 nucleotide sequence fragments encompassing the K76**T** mutations responsible for CQ resistance, while 526 nucleotide sequence fragments of *Pfmdr1* containing the N86**Y** mutations responsible for multidrug resistance and 793 nucleotide sequence fragment of *Pfk13* gene containingN458**Y**, Y493**H** R539**T**, I543**T** and C580**Y**known point mutation responsible of ART resistance were sequenced [17]. The sequences of *Pfcrt, Pfmdr1,* and *Pfk13* found in the study have been deposited in Gene Bank via Bankit http://www.ncbi.nlm.nih.gov/Bankit (Accession # MZ678763-MZ678766 for *Pfcrt*, MZ054306, MZ054305, and MZ678767-MZ678769 for *Pfmdr1,* and MZ151068-MZ151071 for *Pfk13* gene).

### DNA sequence analysis

The DNA sequences were aligned; and population genetic parameters were calculated for each gene separately. Manual editing and alignment of DNA sequences was conducted using SeqMan, EditSeq, and MegAlign modules of the Laser gene computer program [17]. All the parameters were calculated using the computer program DNA Sequence polymorphism v6.12.03 (DnaSP) [21].

### Analysis of *Pfdhfr *&*Pfdhps* genes

Allele specific polymerase chain reaction (AsPCR) assay was performed to investigate the presence of point mutations in *Pfdhfr* and *Pfdhps* gene associated with antifolate resistance as per the published protocol [22].

### Ethics and consent

The Institutional Human Ethics Committee of the ICMR-RMRC Bhubaneswar has approved the study. Before the enrolment, the purpose of the study was explained to the participants in the local language, and verbal consent was obtained for blood sample collection and testing. One consent from the adult patient (above18 year age) and consent for children less than 18-year age from their parents or head of the house hold members in case of no parents, as per ICMR guidelines; written informed consent was obtained from patients or the parents/guardians of children prior to blood collection.

### Data analysis

The data obtained was analysed using Microsoft Excel. Statistical analysis was carried out using *P*-values from a chi-square test for proportions using *P*-value of 0.05, comparing the relationship between different individual mutations, haplotypes, with respect to different geographical regions. The analysis was done using SPSS 20.0 (IBMCorp.2020, Chicago, IL).

## Results

**Fig. 2:**
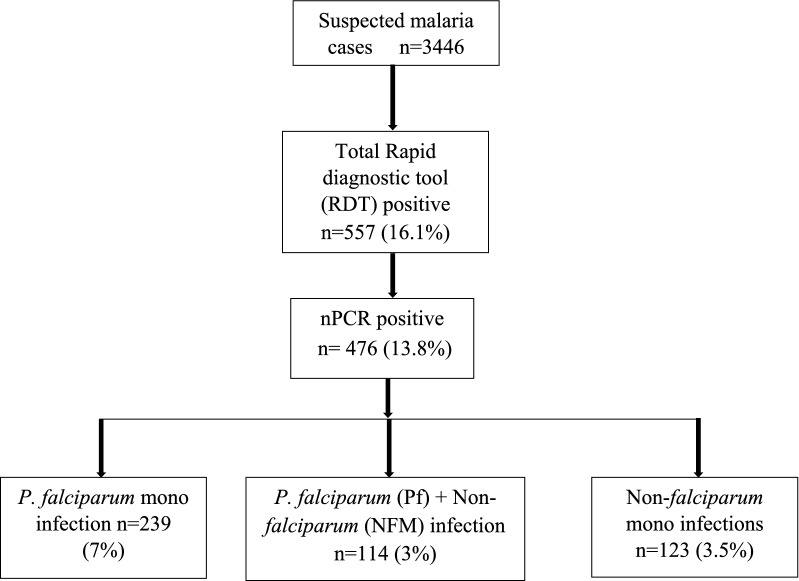
Flow chart of the study sample selection

Of the total 557 RDT malaria positive samples, 476 were found to be positive for malaria by nested PCR. Amongst the PCR-positive samples, 239 were *P. falciparum* mono infection*,* 123 were non-*falciparum* malaria (NFM) (*Plasmodium malariae*: 7, *Plasmodium ovale*: 2 and *Plasmodium vivax*: 114) and 114 were *P. falciparum* mixed with NFM infections. The flow diagram of the selection of *P. falciparum* mono-infection samples used in the present study for molecular analysis of drug resistance genes has been depicted in Fig. 2 and the baseline characteristics of the enrolled cases has been shown in Table 2.

**Table 2:** Baseline characteristics of the enrolled *P. falciparum* mono-infection cases in Odisha (2018 to 2020)

Factors	Variable	*P.falciparum*	Prevalence
		(No)	(%)
Age	< 5	49	21
	6–15	65	27
	≥ 16	125	52
Gender	Male	109	46
	Female	130	54
Clinical	Fever (> 37.0^0^C) Headache, Myalgia	217	91
	Fever (> 37.0^0^C) Headache, Nausea/ Vomiting	22	9
Geographical regions	Eastern Ghat	81	34
	Northern Plateau	95	40
	Central Tableland	46	19
	Coastal Belt	17	7

### Analysis of *Pfcrt, Pfmdr1 *and *Pfk13* gene

PCR–RFLP analysis was performed in 239 *P. falciparum* isolates have shown *Pfcrt* K76**T** point mutation in five isolates (2.1%), *Pfmdr1*N86**Y** point mutation in 8 isolates (3.4%) and no isolate carried *Pfcrt* K76**T** + *Pfmdr1*N86**Y** point mutations.

Single nucleotide mutations identified through DNA sequencing translated to amino acid substitutions in a subset of samples revealed two different types of haplotypes (CV**IET** and CVMN**T**) in isolates having *Pfcrt* 72-76 point mutation, the primary determinant of chloroquine (CQ) resistance, while analysis of wild type (*Pfcrt* K76) samples shown CVMNK haplotype. Of the two different kinds of haplotypes detected in mutant samples during the survey, both the haplotypes (CV**IET** and CVMN**T**) were found in the *P. falciparum* isolates collected from the Eastern Ghat (Raygada and Kandhamal), while the CV**IET** was found in Sundargarh district of Northern Plateau region and CVMN**T** in Bargarh district of Central Tableland region as shown in Table 3. SVMNT haplotype was not reported in the recent study. Similarly, sequencing of the *Pfmdr1* mutant isolates detected by PCR–RFLP showed N86**Y** and N86**Y**/Y184**F** point mutations. There was no significant difference (χ^2^ = 6; P > 0.05) in the distribution of *Pfcrt* and *Pfmdr1* associated with chloroquine drug resistant genes in the four different geographical regions of the state as shown in Table 3.

**Table 3:** Table showing prevalence of *Pfcrt* and *Pfmdr1*gene haplotypes found in four different geographic regions

Geographical region	Northern plateau	Prevalence	Central table land	prevalence	Coastal belt	Prevalence	Eastern ghat	prevalence	*P*-value	Chi square value	Degree of freedom (df)
Haplotypes	(N)	%	(N)	%	(N)	%	(N)	%		χ^2^	df
CVMNK	0	0	3	17.6	3	17.6	6	35.2	0.199	6	4
CVIET	1	5.8	1	5.8	0	0	1	5.8	0.199	6	4
CVMNT	0	0	1	5.8	0	0	1	5.8	0.199	6	4
N86Y	2	25	1	12.5	1	12.5	1	12.5	0.199	6	4
Y184F	0	0	0	0	0	0	1	12.5	0.199	6	4

Though none of the validated or established mutations associated with artemisinin resistance detected in *P. falciparum* isolates subjected for DNA sequencing, six synonymous mutations that were not coding any proteins but changes only in change of nucleotides i.e. A160C, A208G, C210G, T211G, T212G, A251G were reported in this study, indicating the absence of *P. falciparum* genotype (*Pfk13*) associated with resistance to artemisinin in Odisha at present. The population genetic parameters for all the three genes responsible for anti-malarial resistance are displayed in Table 4. While the haplotype diversity was almost similar in all the three genes, the nucleotide diversities, as measured independently by theta (ϴ) and Pi (π), were variable across the three genes. Whereas relatively higher nucleotide diversities were found in the *Pfcrt* gene for both the parameters theta (ϴ) and Pi (π), the values were found to be lower in the *Pfmdr1* and *Pfk13* genes. The test of neutrality as measured by Tajima *D*, Fu and Li’s *D,* and Fu and Li’s *F* tests were not statistically significantly deviated from the model of neutral expectation in any of the three genes.

**Table 4:** Details of *P. falciparum*K-13 propeller gene (*Pfk13*), *P. falciparum* chloroquine-resistance transporter (*Pfcrt*) and *P. falciparum* multi drug resistance-1(*Pfmdr-1*) nucleotide fragments and population genetic parameters in *P. falciparum* field isolates of Odisha, India

Gene (Size of the Reference Sequence)	*Pfcrt* (3107 bp)	*Pfmdr-1* (4382 bp)	*PfK13* ( 2417 bp)
Nucleotide positions	403,222–406,317	957,890–962,149	1,724,817–1,726,997
Total number of isolates	17	8	9
Size of the fragment (nucleotide)	212	526	793
Total number of polymorphic (segregating) sites	4	2	6
Total number of singleton mutations	1	1	2
Total number of SNPs	4	2	6
Total number of haplotypes	5	3	5
Haplotype diversity	0.64	0.679	0.722
Nucleotide diversity π	0.00472	0.00156	0.00266
Nucleotide diversity (ϴ)	0.00558	0.00147	0.00278
Tajima’s *D*	− 0.47378	0.24178	− 0.19073
Fu and Li’s *D*	0.23149	− 0.14931	0.26719
Fu and Li’s *F*	0.04712	− 0.06487	0.17557

### Analysis of *Pfdhfr* and *Pfdhps* genes

A total of 239 *P. falciparum* infected blood samples were analysed for mutations in six codons of the *Pfdhfr* gene (A16**V**, C50**R**, N51**I**, C59**R**, S108**N/T** and I164**L**) and five codons of the *Pfdhps* gene (S436**F/A**, A437**G**, K540**E**, A581**G** and A613**S/T**) to assess the level of antifolate drug pressure. Out of 239 samples, 232 were PCR positive for *Pfdhfr* and 221 for *Pfdhps, w*hile PCR could detect both *Pfdhfr* and *Pfdhps* genes in 119 (49.7%) of the samples. The *Pfdhfr* C59**R** mutation was found to be most prevalent (N = 97, 41.8%), followed by the C50**R** mutation (N = 93, 40.8%) and S108**N** mutation (N = 91, 39.2%), No isolate had the S108**T** mutation, while the N51**I**, I164**L**, and A16**V** mutations were found in 17.2% (N = 40), and 3.4% (N = 8) and 9.05% (N = 21) of the isolates respectively as shown in Table 5. There was no significant difference between χ^2^ = 8, P-value = 0.238 for A16**V**, N51**I** and I164**L** and, χ^2^ = 12, *P*-value = 0.213 for C50**R**, C59**R** and S108**N** individual amino acid mutation of the *Pfdhfr* gene in the four different geographical regions of the state as shown in Table 5. The wild-type *Pfdhfr* sequence (ACNCSI) at all six codons was prevalent in 49.6% (N = 115) of the isolates. Amongst the total isolates 26 (11.2%) had a single mutation, 19 (8.2%) had double, 40 (17.2%) had triple, 23 (9.9%) had quadruple, 8 (3.4%) had quintuple and 1 (0.43%) had sextuple mutation. The most frequent triple mutation sequence was A**R**N**R**S/**N**I (N = 18, 7.8%) A**RIR**S/SI (N = 17, 7.3%) and the quadruple mutation sequence was A**RIR**S/**N**I (N = 12, 5.2%) **VR**N**R**S/**N**I (N = 11, 4.7%). In our sample (N = 232) total 13 different haplotypes have been observed in the *Pfdhfr* gene in four different geographical regions of the state as shown in Table 6. There was no significant difference (as P-value > 0.05) in 13 haplotypes of *Pfdhfr* gene with respect to four different geographic regions of the state i.e. Northern Plateau; χ^2^ = 78, P-value = 0.294, Eastern Ghat; χ^2^ = 117, P-value = 0.261, Central Tableland; χ^2^ = 39, P-value = 0.336, and, Coastal Belt; χ^2^ = 26, P-value = 0.353, as shown in Table 7.

**Table 5:** Regional distributions of drug-resistance molecular markers (individual Amino acid codon mutations) in *P. falciparum* isolates in Odisha, India (Period: 2018–2020)

Genes	*Pfcrt K76T*	*Pfmdr1 N86Y*	*Pfdhfr*						*Pfdhps*						
Geographical regions	K76**T**	N86**Y**	A16**V**	C50**R**	N51**I**	C59**R**	S108**N**	I164**L**	S436**F**	S436**A**	A437**G**	K540**E**	A581**G**	A613**S**	A613**T**
Eastern ghat	2	1	16	62	35	66	59	6	26	34	17	22	31	20	23
Northern plateau	1	2	5	26	5	26	20	2	10	17	8	22	8	9	8
Central table land	2	1	0	3	0	3	8	0	5	2	3	4	3	2	1
Coastal belt	0	1	0	2	0	2	4	0	3	2	2	2	2	1	2
Total	5	5	21	93	40	97	91	8	44	55	30	50	44	32	34
χ2	8	4	8	12	8	12	12	8	12	8	12	8	12	12	12
Degree of freedom (df)	6	3	6	9	6	9	9	6	9	6	9	6	9	9	9
*P*-value	0.238	0.261	0.238	0.213	0.238	0.213	0.213	0.238	0.213	0.238	0.213	0.238	0.213	0.213	0.213

**Table 6:**
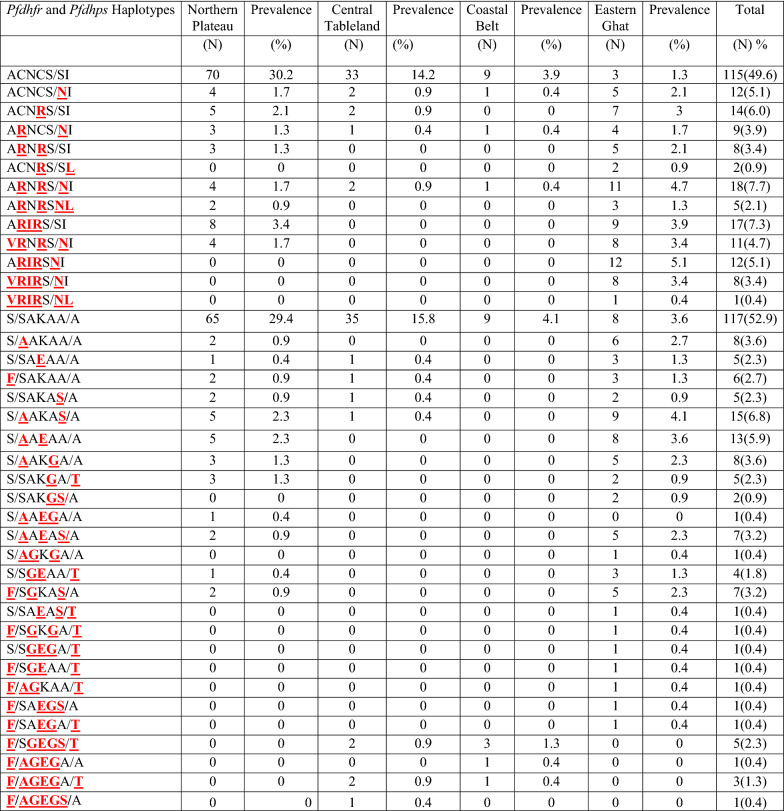
Regional distribution of different *P. falciparum Pfdhfr* (N = 13), and *Pfdhps* (N = 26), haplotypes detected in the state of Odisha during the study period 2018–2020.

N: Number of isolates analysed, mutated codons are red coloured, bold and underlined. (*N*   number of haplotypes, % = prevalence of haplotypes)

**Table 7:** Distribution of *Pfcrt* (N = 3), *Pfmdr1* (N = 2), *Pfdhfr* (N = 13), *Pfdhps* (N = 26) haplotypes in four geographical regions of the state Odisha (period: 2018–2020)

Geographical regions	Northern Plateau			Central Table land			Coastal Belt			Eastern Ghat		
Haplotypes	χ2	df	*P*-value	χ2	df	*P*-value	χ2	df	*P*-value	χ2	df	*P*-value
*Pfcrt*												
CVMNK	15	12	0.241	15	12	0.241	15	12	0.241	15	12	0.241
CVIET	6	4	0.199	6	4	0.199	6	4	0.199	6	4	0.199
CVMNT	6	4	0.199	6	4	0.199	6	4	0.199	6	4	0.199
*Pfmdr1*												
N86Y	6	4	0.199	6	4	0.199	6	4	0.199	6	4	0.199
Y184F	8	6	0.238	8	6	0.238	8	4	0.199	8	6	0.238
*Pfdhfr*												
for 13 alleles	78	72	0.294	39	36	0.336	26	24	0.353	117	108	0.261
*Pfdhps*												
for 26 alleles	130	125	0.326	78.000	75.000	0.384	78	75	0.384	182	175	0.343

*N* number of haplotypes observed in the study, χ2 = Chi square test, *df *degree of freedom

*P-value =  < 0.05 significant*

Of the 221 samples PCR positive for *Pfdhps,* 117 (52.9%) had the wild-type sequences (SAKAA) at all five codons. The maximum number of mutations were found at codon S436**A** (N = 59, 26.7%), followed by A613**S** (N = 39, 17.6%), K540**E** (N = 38, 17.2%), A581**G** (N = 31, 14.0%), S436**F** (N = 28, 12.7%), A437**G** (N = 26, 11.8%) and A613**T** (N = 23, 10.4%) as shown in Table 5. There was no significant difference (as *P*-value > 0.05) between S436**F**, A437**G**, A613S**,** A581**G,** A613**T** (χ^2^ = 12, P-value = 0.213) and for S436**A**, K540**E** (χ^2^ = 8, *P*-value = 0.238) of the *Pfdhfr* gene codons in the four different geographical regions of the state as shown in Table 5. In comparison to single (N = 24, 10.9%), triple (N = 21, 9.5%), quadruple (N = 6, 2.7%), quintuple (N = 6, 2.7%), and sextuple (N = 4, 1.8%) mutations, double mutations in *Pfdhps* gene had more prevalent (N = 43, 19.5%) as shown in Table 6. Amongst the double mutations, the sequences with S/**A**AKA**S/**A (N = 15, 6.8%) and S/**A**A**E**AA/A (N = 13, 5.9%), and amongst the triple mutations the sequences with S/**A**AK**G**A/A (N = 8, 3.6%), S/**A**A**E**A**S/**A (N = 7, 3.2%), **F/**A**G**KA**S/**A (N = 7, 3.2%), **F/**S**GEG**A/**T** (N = 5, 2.3%) were common, as shown in Table 6. There was no significant difference (as P-value > 0.05) in 26 haplotypes of *Pfdhps* gene with respect to four different geographic regions of the state i.e. Northern Plateau; χ^2^ = 130, P-value = 0.326, Eastern Ghat; χ^2^ = 182, P-value = 0.343, Central Tableland and Coastal Belt; χ^2^ = 78, *P*-value = 0.384, as shown in Table 7.

### Plasmodium falciparum* dhfr-dhps* two-locus mutation analysis

The *P. falciparum Pfdhfr-Pfdhps* two-locus mutation analysis carried out in 119 (49.79%) isolates have revealed 3 different genotypes in Coastal Belt, 5 in Central Tableland, 29 in Northern Plateau and  ≥ 40 in Eastern Ghat regions. However, no isolate with *Pfdhfr* triple (N51**I**/C59**R**/S108**N**) mutation in combination with *Pfdhps* double (A437**G**/K540**E**) mutation, a useful predictor of SP treatment failure, was found in the studied sample.

## Discussion

The present study conducted during 2018–2020 has demonstrated a low prevalence (2.1%) of *Pfcrt* K76**T** mutation associated with resistance to CQ in *P. falciparum* isolates circulating in Odisha. Moreover, the same low percentage of mutation has also been detected for *Pfmdr1* N86**Y** (3.4%). In contrast, a high prevalence of *Pfcrt* K76**T** (67.5%) and *Pfmdr*1 N86**Y** (80%) was observed in another study conducted before CQ withdrawal from the state during 2008–2010 [17]. The result obtained is similar to the observations made in Malawi, Tanzania, Mozambique, Northern Uganda, Saudi Arabia [23–27], but in contrast to southern Benin [28] and in other parts of India reported recently [29–32]. The present study, although limited to a small number of samples, indicates not only the presence of three types haplotypes (CVMNK: wild type, CV**IET** mutant type: believed to be of Southeast Asian origin, CVMN**T** mutant type: believed to be of African Origin) but also inform the high genetic diversity present in field isolates of *P. falciparum* for CQ drug-resistant genes in Odisha, India. Interestingly, the wild type CVMNK haplotype of the *Pfcrt* gene was found in 76.47% of the isolates, which is in contrast to findings from other studies in Indian *P. falciparum* as documented in earlier studies [33]. It is also argued that Odisha might have served as the epicentre for the distribution of chloroquine-resistant *P. falciparum* parasites to other parts of India [34, 35].

More than 200 non-synonymous mutations have been identified in K13 protein from *P. falciparum* strains in different malaria endemic countries and 50 of them are shown to be associated with ART treatment failure [36]. Studi**e**s conducted in India have identified fourteen K13 mutations in K189**T**, F446**I**, A481**V**, G533**A/S**, R539**T**, S549**Y**, R561**H**, A578**S**, M579**T**, G625**R**, N657**H**, N672**S**, A675**V** and D702**N** [37–39] and two of them (R539**T**, G625**R**) are shown to be associated with ART resistance [37]. No non-synonymous mutations have been observed during the present study despite occurrence of silent/synonymous mutations. DNA sequence polymorphism study that not only informs distribution of different haplotypes, but also the evolutionary potentiality of mutation in the drug-resistant genes that can directly translate to molecular epidemiology of human diseases like malaria. Several studies employing this methodology on the three genes of interest for malaria public health have been conducted worldwide, which has immensely helped in determining intervention through therapeutic measures and change in drug policy in different countries [40–44].

Sequential accumulation of S436**F/A**, A437**G**, K540**E**, A581**G**, A613**S/T** mutations in *Pfdhps* [45]and A16**V**, C50**R**, N51**I**, C59**R**, and S108**T/N** mutations in *Pfdhfr* [46] leads to the development of resistance to sulphadoxine and pyrimethamine respectively in *P. falciparum* isolates [47]. The primary mutation being A437**G**/ K540**E** in *Pfdhps* and S108**N**/ C59**R** in *Pfdhfr* as per the findings from different malaria endemic regions of the world including India [22, 48–50].The high proportion of mutation at codon C59**R** (41.4%), C50**R** (38.4%) and S108**N** (32.8%) mutations in the *Pfdhfr* gene than at codon S436**A** (26.7%),A613**S** (17.6%) and K540**E** (17.3%) mutations in *Pfdhps* gene indicate that these are the key point mutations and further the overall low prevalence of point mutations in *Pfdhps* (47.05%) gene sequence compared to *Pfdhfr* (50.4%) confirms that the mutations associated with parasite resistance to SP appeared earlier on the *Pfdhfr* than those affecting the *Pfdhps* [28]. The prevalence of single, double, triple, quadruple or quintuple mutation in *Pfdhfr* and *Pfdhps* observed in this study reflects the current level of sensitivity of *P. falciparum* to SP. Moreover, mutation at codon C59 and S108 along with codons A16, C50 and N51 in *Pfdhfr* (~ 38%) and codon A437 and K540 along with codons S436, A581 and A613 in *Pfdhps* (~ 24%) during the present study strongly predicts the decreasing treatment response as reported earlier in Western and Central Africa [51–53]. Out of the 12 *Pfdhfr* mutant genotypes found in the *P. falciparum* isolates in the state, 3 mutant genotypes (ACNCS/**N**I, ACN**R**S/SI, A**R**NCS/**N**I) have been reported earlier in India including Odisha [22], while 8 mutant genotypes (A**R**N**R**S/SI A**R**N**R**S/**N**I ACN**R**S/S**L** A**RIR**S/SI A**R**N**R**S/**NL**, A**RIR**S/**N**I, **VRIR**S/**N**I, **VRIR**S/**NL)** have been found for the first time in the state. Similar is the situation in the case of *Pfdhps* gene, in which S/**A**AKA**S/**A, S/**A**A**E**A**S/**A, **F/**A**G**KA**S/**A, **F/**S**GEG**A/**T, F/**S**GEGS/T, F/AGEG**A/**T** and **F/AGE/G**AA genotypes have been detected for the first time in the state in addition to S/**A**A**E**AA/A and S/SAK**G**A/**T** genotypes reported from India including Odisha. Prevalence of such unique multiple mutations in *Pfdhfr* as well as *Pfdhps* in the state indicates emergence of resistance to SP, the currently used partner drug of ACT in the state, as observed earlier in Kenya, Thailand and Vietnam [22]. But, absence of linked N51**I**-C59**R**-S108**N** codons in *Pfdhfr* and A437**G**-K540**E**codons in *Pfdhps* indicates that *P. falciparum* isolates circulating in this part of the country have not developed RIII (highest) level of resistance [54]. Similarly, absence of A16**V**-S108**T** mutation in *Pfdhfr* responsible for cycloguanil resistance in the present study might be because cycloguanil-proguanil has not yet been introduced for the treatment of malaria in India [7].

## Limitation of the study

There are some limitations that should be considered when interpreting the findings of the present study. First, the total number of the collected *P. falciparu*m isolates was small and disproportionate to different geographical region. Second, the molecular analysis (DNA sequencing) has been done in a subset of samples instead of total sample. Third, the copy number of *Pfmdr1* has not been analysed.

## Conclusion

This was the first molecular study carried out in the state of Odisha (India), after a gap of ten years of CQ withdrawal, focusing on mutations of *Pfcrt* and *Pfmdr*1 genes strongly associated with CQ, *Pfkelch13* associated with ART, and *Pfdhps* and *Pfdhfr* genes strongly associated with resistance to SP, the partner drug used with ACT in the current drug policy. This study showed low prevalence of resistance to marker CQ that dramatically contrasted with our earlier study in the state. This study found an absence of *Pfkelch13* mutations associated with ART resistance in *P. falciparum* isolates. However, the prevalence of triple, quadruple, quintuple and sextuple mutated *Pfdhfr*-*Pfdhps* genotypes sounds an alarm and, therefore, continuous molecular and in vivo monitoring of ACT is recommended for ensuring proper malaria control.

## Data Availability

All data generated or analysed during this study are included in the article. The nucleotide sequences, except *Pfdhfr* and *Pfdhps*, generated have been deposited in Gene Bank via Bankit http://www.ncbi.nlm.nih.gov/Bankit/ and are available in the database under the accession numbers as indicated in the text.
